# The Geopolitics of Health Science Research: Comment on "The Roles of Regional Organisations in Strengthening Health Research Systems in Africa: Activities, Gaps, and Future Perspectives"

**DOI:** 10.34172/ijhpm.2022.7516

**Published:** 2022-12-21

**Authors:** Pádraig Carmody

**Affiliations:** ^1^Department of Geography, Trinity College Dublin, Dublin 2, Ireland.; ^2^School of Tourism and Hospitality, University of Johannesburg, Johannesburg, South Africa.

**Keywords:** COVID-19, Geopolitics, Health Research, Africa

## Abstract

African development is defined by a number of meta-trends, including climate disruption, digitalisation, informalisation, regionalisation and most recently the impacts of the coronavirus disease 2019 (COVID-19) pandemic. The paper under consideration here is informed primarily by two of these: regionalism and the COVID-19 pandemic. Africa, or at least parts of it, have been severely affected by pandemics in recent decades. At the same time deepening regionalisation allows for more coordinated and effective actions to mitigate their worst effects. However, to date, regional integration efforts have not generally delivered desired results, and in the area of Health Science Research (HSciR) specifically, which is the area of focus for this paper. This important paper considers the nature of current activities in relation to health research by regional organizations on the continent. It provides a baseline study and incipient manifesto for increased effectiveness and greater contribution in the area of HSciR on the continent.

 Regionalism is an increasingly important policy area and subject of academic study in Africa. In the theory of regionalism there are generally two main identified approaches: open and developmental regionalism. Open regionalism prioritises economic liberalisation to integrate the region more deeply into global structures and flows, whereas developmental regionalism seeks better coordination between participating countries through planning to enhance outcomes. The paper^1^ under consideration here arguably fits better with the second approach. It seeks to elucidate current “roles, challenges and opportunities” for regional organisations in health science research (HSciR) on the continent. This is an extremely important and topical issue. The paper is very well-researched and written and has many important insights and suggestions about how such bodies can make enhanced contributions to the societies which they serve.

 Amongst the most important points the paper makes is that national authorities can learn from regional experience and organisations through closer coordination and awareness. It also argues that health research can be a spur to economic development. A political economy analysis of the current configuration of regional organisations in this area might examine why they have generally had less impact than might be envisaged or desired by those who set them up or are currently involved in their functioning. One of the issues is perhaps health science systems’ extraversion. Some respondents noted that dependence on external finance is a problem and indeed some of the organisations were only set up with donor support. For example some regional organisations’ headquarters, such as the Africa Centres for Disease Control (Africa-CDC) and the African Union’s were funded by the Chinese government. In addition to promoting improvements in health these are examples of China’s soft power on the continent, as aid is a branch of foreign policy. Limited joined up thinking, policy and planning may be partly as a result of the importation of foreign models (the American Center for Disease Control seems is perhaps the model for the African version) and external orientation or responsiveness as a result of budgetary dependence. There is then a political economy of (in)effectiveness beyond technical questions of coordination and financing for example. Interesting the paper notes that it is “Africa-CDC and WHO [World Health Organization] regional offices were the regional organisations which appeared to have the most authority and leadership in this area across the continent.” The WHO is a global, rather than a regional one and there have been concerns expressed in recent years about the extent of some funders outsized influence in the organisation, such as China despite its relatively low level of funding support. “Nearly all informants cited dependence on foreign and external funds as important barrier for ownership and local benefits of HSciR on the continent.”

 The paper however also details the successes of regional organisations in this area. For example the fact that the West African Health Organisation has developed a single medicines registration process for all of its 15 member states. However there appear to be a variety of institutional voids on the continent with “few organisations…coordinating across remits of multiple stakeholders at continental level,” for example. This and other examples highlighted in the paper suggest there is substantial room for enhanced outcomes through better coordination and resourcing; an issue highlighted in the recent report on the reform of the African Union. Perhaps somewhat surprisingly there was no regional organization whose core mandate is HSciR. However there are institutions within regional economic communities with core mandates that include HSciR, such as the East African Health Research Commission, which is an East African Community institution, and West African Health Organisation which is an organ of the Economic Community of West African States.

 In terms of financing two issues were highlighted by respondents – the relative lack of funding of HSciR by development finance institutions and the private sector. In a sense this is perhaps not surprising. While health became an area of geopolitical competition amongst the “great” powers during the coronavirus disease 2019 (COVID-19) pandemic,^2^ with some accusing China of pursuing “mask diplomacy”^3^ through donations of personal protective equipment for example, thereby occluding its strategic motivations, the focus of competition still appears to be on “hard infrastructure.” At the most recent G7 summit in 2022 leaders announced substantial funding for an infrastructure initiative which would compete with China’s “Belt and Road.” This was a response to China’s global developmental approach, which has been seen to deliver concrete infrastructure across world regions, with long-lasting developmental effects, including in many cases debt. Nonetheless hard infrastructure is often an attractive proposition for incumbent political elites because it creates jobs and boosts economic growth in the short-term, thereby sometimes aligning with political business cycles. HSciR however will only yield (politic) dividends in the longer term, and has indirect impacts and diffuse benefits. Many respondents in the study reported that “the commitment of membership to regional work is necessary because state inaction or state action that does not align with regional priorities can hinder progress.” Given the often fragile nature of “political settlements” in Africa, regime maintenance is often a, or the top state priority. This may leave regional organisations between the Scylla of state power and the Charybdis of external domination. This may account for the weaknesses identified in the paper around the need to clarify roles of regional organisations in this space, the ways in which they “build, support or participate in networks of HSciR” and “lack of development of regulatory institutions for health research or science,” amongst others. The authors advocate that the economic growth frame may be used to support the case for HSciR. This may improve the chances of getting more traction in this area, however there are also broader political economy considerations at play.

 This paper serves an important function by providing a baseline study of the role of regional organisations in HSciR in Africa. This is an important area where there may be important coordination gains enabling a variety of social, individual and economic benefits. However, there are a variety of issues and choices to be considered. The first, highlighted in this response, is the political economy of regional organisations and HSciR. A second is the socio-economic cost benefit analysis and trade-offs implied. Health is a vitally important area, perhaps the most important in terms of human welfare. This is increasingly recognized through multi-dimensional definitions and understandings of poverty and their increased usage. However public resource deployment necessarily entails opportunity costs and trade-offs. Increased resourcing, including institutional redesign and coordination, necessarily imply this. The authors note the economic growth frame as a way to persuade political leaders to more substantially resource this research sector. However, African governments and regional organisations face many competing priorities, such as peace and economic development for example. An argument might also be made for better coordination of economics or agricultural research and policy coordination across the continent for example. Other studies may be needed in these and other areas in order for public decision makers to make more informed choices about resourcing. Of course a social development perspective would argue that improved HSciR will inform better policy and health outcomes, which will contribute to stronger economies and potentially help drive a virtuous development circle. This is then not an argument not to properly fund this vitally important area for human welfare and development. Rather this study is perhaps one of several that are needed to bring to light different institutional voids and gaps and ways in which they might be overcome. There may be a strong case for increased resourcing and coordination in the area of HSciR for regional organisations, particularly as they may be more insulated from domestic political imperatives. Of course this may make generating the political will for such a shift more difficult.

 Another area which might be of interest for further research and study, might be a comparison of the African case with those of other world regions to see both what they might learn from this continent’s experience, both positive and negative, and if there might be potentially transferrable experience to Africa. Is HSciR often a priority of regional organisations globally, and if so why not? Is HSciR generally nationally oriented, given extreme differences in disease prevalence across regions and sub-regions? What areas make sense to coordinate on and which are better left to national levels? Given generally weak HSciR research capacities at national levels in Africa can regional organisations play a more important role by compensating for this and insulating from political pressures? These are some of the questions which this important paper provokes. I hope it will be widely read and that it will spur further studies which might help us answer some of these questions.

## Ethical issues

 Not applicable.

## Competing interests

 Author declares that he has no competing interests.

## Author’s contribution

 PC is the single author of the paper.
